# Subject-specific Functional ROIs Enhance Reliability in Language FMRI

**DOI:** 10.1007/s00062-025-01534-3

**Published:** 2025-07-09

**Authors:** Julia My Van Kube, Luisa Katrin Thomas, Peter Dechent, Christian Heiner Riedel, Nicole E. Neef

**Affiliations:** 1 Department of Diagnostic and Interventional Neuroradiology, University Medical Center Göttingen, Robert-Koch-Straße 40, 37075 Göttingen, Germany; 2 Department of Cognitive Neurology, MR Research in Neurosciences, University Medical Center Göttingen, Robert-Koch-Straße 40, 37075 Göttingen, Germany

**Keywords:** Functional magnetic resonance imaging, Language mapping, Functional regions of interest, Individual-subject analyses

## Abstract

**Purpose:**

Functional MRI can be used to identify individual language-sensitive brain regions in the setting of presurgical diagnostics to improve functional postoperative outcome. In this study, a proven language task was adapted into German and tested with regard to its effectiveness, robustness and reliability in a time frame appropriate for the clinical setting. In addition, two different analysis approaches were compared to address the problem of arbitrary statistical thresholds commonly used in the clinical routine to derive contrast maps.

**Methods:**

On two different days, 24 healthy volunteers were examined in a 3T MRI, whereby the task was run twice in each session. The fMRI included two conditions in a block design, reading of sentences and reading of pronounceable nonword lists. We quantified brain activity by using subject-specific, functionally defined ROIs on the one hand and standardized, anatomically defined ROIs on the other. We then tested, whether the two different analyses indicated robust activation of language-sensitive brain regions, and whether effect sizes were reliable across sessions.

**Results:**

Subject-specific functional ROIs as well as anatomical ROIs led to significant positive effect sizes in the major language sensitive regions of the left hemisphere. However, subject-specific functional ROIs resulted in significantly larger effect sizes and a higher reliability in comparison to anatomical ROIs.

**Conclusion:**

The choice of analysis method has a significant impact on the result. For paradigms with short measurement times and little signal change as common in clinical routine, it is highly recommended to use the subject-specific functional ROIs approach.

**Supplementary Information:**

The online version of this article (10.1007/s00062-025-01534-3) contains supplementary material, which is available to authorized users.

## Introduction

Functional MRI is an advanced imaging technique that can reduce morbidity in brain tumor resection [1]. It provides valuable insights into the functional organization of the brain, aiding in identifying eloquent brain areas in the context of presurgical planning for conditions such as brain tumors, vascular malformations, or epilepsy. With fMRI clinicians can map brain areas associated with cognition and language functions and determine functional language lateralization. Maintaining language functions during surgical procedures significantly contributes to improved patient outcomes and quality of life. However, several factors limit the use of fMRI in routine clinical practice, including time constraints, patient cooperation, task variability, and data analysis issues. fMRI time is scarce and robust and reliable language maps are difficult to generate [2]. Crucially, extensive neurobehavioral fMRI experiments have led to a paradigm that results in a robust and reliable stimulation of language-sensitive brain areas [3, 4]. But the measuring times were often longer than 20 min and reliability tests relate only to English stimuli [5]. In this study, we adapted the proven paradigm into German and tested its effectiveness, robustness and reliability.

The effectiveness of an fMRI paradigm in an individual subject not only depends on stimulation intensity and duration, but also on statistical thresholding [6]. Statistical thresholding helps separate signal from noise, and fMRI analysis are based on multiple comparison correction methods, such as the false discovery rate or family-wise error rate to address the issue of inflated Type I errors due to massive multiple statistical testing [7, 8]. The choice of threshold influences the identification of brain regions activated during a specific task and the reliability of the respective result [9]. It is critical and involves a trade-off between sensitivity and specificity. A threshold that is too lenient may result in detecting spurious activations, while a too conservative threshold may lead to missing genuine activations [10]. The determination of an appropriate threshold can be somewhat subjective, and clinicians may use a combination of statistical and practical considerations to arrive at a reasonable activation map and ultimately an assessment of language localization and lateralization [11].

One strategy to overcome subjective statistical thresholding to quantify activity and calculate language laterality indices is the use of a predefined set of ROIs [12]. A priori defined ROIs reduce the need for statistical thresholds, as the averaged change in signal across all voxels within a given ROI determines whether there was positive, negative or no activity. Several approaches exist to select ROIs that cover language-sensitive brain areas. Here we compare the effectiveness of anatomical (a)ROIs derived from an automated parcellation of the cortical surface [13] and functional (f)ROIs derived from a study in which a group of healthy participants performed a similar language task [4, 5]. The selection of fROIs was not only driven by group activation patterns from language-stimulation, but considered in addition the subject-specific occurrence of brain activity in a given fROI, creating individual masks for each subject. By contrast, the selection of anatomical ROIs was motivated from a meta-analysis synthesizing the topology of language activity in the brain [14].

More specifically, on one hand, the selection of aROIs was motivated by a meta-analysis synthesizing the topology of language activity in the brain [14]. On the other hand, the selection of fROIs is based on group activation patterns from language-stimulation from which group-constrained functional partitions were derived [4, 5]. Within these functional partitions, the subject-specific occurrence of brain activity was used to create individual masks for each subject. This approach takes into account inter-individual anatomical differences in language area localization, thereby increasing sensitivity and functional resolution of fMRI analysis [15–17]. This is particularly valuable in the clinical setting, where it can help identify small BOLD signal changes associated with cognitive tasks [18], reducing the need for repeated examinations and minimizing the burden on patients and resources.

We hypothesize that aROIs are less sensitive and less reliable to capture language-sensitive brain activity than subject-specific fROIs. Our main aim was to test the effectiveness, robustness and reliability of our newly generated stimuli and to test the effect of two different analysis approaches.

## Methods

### Participants

We examined 24 healthy volunteers (16 females) between the ages of 18 and 65 years (mean 33 years). Besides contraindications for MRI examination exclusion criteria included underlying neurological or psychiatric disorders, drug-treated chronic diseases, anamnestic drug abuse and current pregnancy or breastfeeding. All subjects were native speakers of German and had normal or corrected-to-normal vision. According to the Edinburgh Inventory [19] 19 participants were right-handed, four were ambidexter and one participant was left-handed. The study was approved by the Ethics committee of the University Medical Center Göttingen and was in accordance with the guidelines of the Declaration of Helsinki. All participants gave written informed consent and obtained an expense allowance for taking part in this study.

### Language Mapping Paradigm

The stimuli were visually projected on a back projection screen positioned at the end of the magnetic bore using a video projector outside the magnet. Subjects saw the presentation via a mirror attached to the head coil. Outside the scanner room, a Windows Computer using the software ‘Presentation Version 23.0’ (https://www.neurobs.com) was connected to the video projector. Participants conveyed their responses via use of a button box with their left and right hand.

**Fig. 1:**
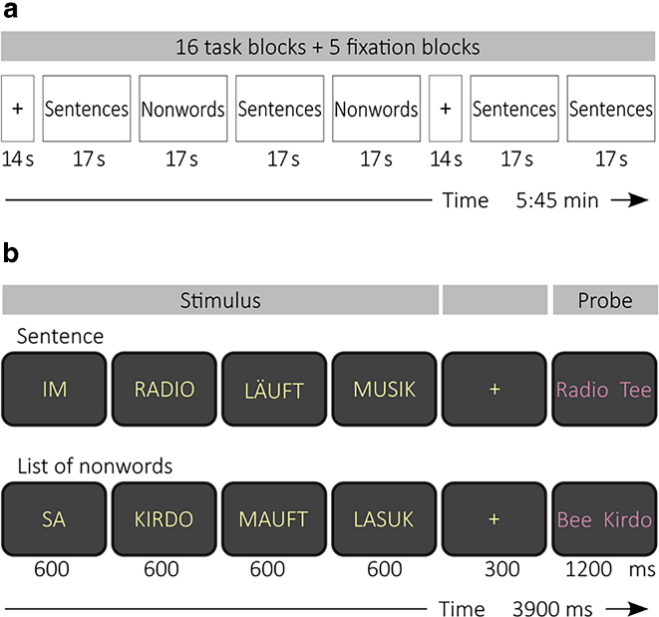
Schematic illustration of (**a**) task and (**b**) example trials. The inter-trial-interval was 300 ms

We adopted a paradigm from a previous series of studies [3–5]. German sentences and lists of pronounceable nonwords were presented in a block design (Fig. 1), with each block containing four trials of one condition. In order to create equal conditions, nonwords were matched to the sentence stimuli in terms of key linguistic parameters, such as syllable count, phonetic complexity, and phonotactic features. The pseudowords were generated with the software Wuggy [20]. The total number of sentences and nonword lists was balanced with 32 trials per run per condition. The participants were instructed to read the words silently. Each word of a sentence or a nonword list was consecutively shown on the screen followed by a decision task in which the participants had to report via a button press which one of two words shown was part of the current trial. One run of the paradigm consisted of 16 blocks and 5 periods of rest and lasted in total 5 min and 45 s. The participants were examined at two different timepoints. During each session two runs of the paradigm were measured. The order of runs was randomized for each participant. The participants were trained on the paradigm with a different set of stimuli in advance of each session.

### Data Acquisition

The measurements of this study were acquired as part of a larger MRI study with further paradigms. MRI imaging was conducted on a Siemens 3 T Magnetom Prisma Fit with a 64-channel, phased array head coil at University Medical Center Göttingen. The participants were examined in a supine position. Functional data was acquired with gradient-echo EPI sequence (TR = 1500 ms, TE = 30 ms, flip angle = 72°, parallel acquisition factor = 2, simultaneous multi-slice factor = 3, FoV = 208 mm, 63 slices) with an in-plane resolution of 2 × 2 mm^2^, a distance factor of 10% and a 104 × 104 acquisition matrix. We acquired 235 volumes per run, with an acquisition time of 6 min 2 sec. As a whole-brain anatomic reference a high-resolution 3D T1-weighted turbo fast low-angle shot sequence (TR = 1900 ms, TE = 2.26 ms, TI = 900 ms, flip angle = 9°, FoV = 256 mm, 7/8 Fourier phase encoding) with a voxel size of 1 × 1 × 1 mm^3^ was used.

### Regions of Interest

**Fig. 2:**
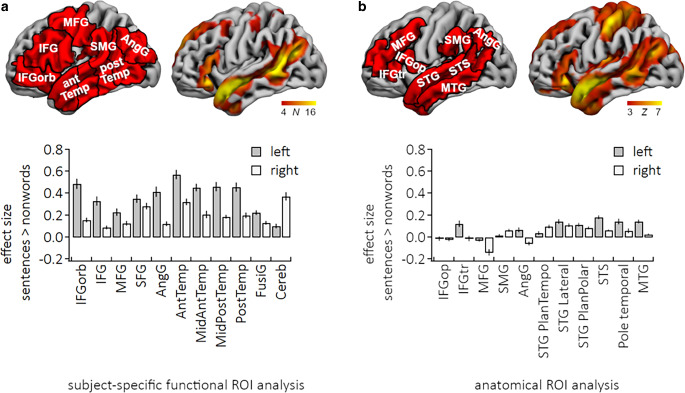
Language-related brain activity. (**a** 3D rendered semi-inflated surfaces of the left hemisphere of the brain with group-constrained functional partitions (red, left) and a probabilistic overlap map for the sentence > nonword contrast for 24 subjects (right). The overlap map indicates points of high inter-subject overlap and the distribution of individual activations around these high overlap points, **b** 3D rendered brain surfaces with anatomical partitions (red, left) and a random-effects map for the sentence > nonword contrast in 24 subjects (right). Bar plots show group averages ± standard error of the effect size for sentences > nonwords for (**a**) subject-specific functional and (**b**) anatomical ROIs. (Abbreviations: *AngG* angular gyrus, *AntTemp* anterior temporal lobe, *Cereb* Cerebellum, *IFG* inferior frontal gyrus, *IFGorb* IFG pars orbitalis, *IFGop* IFG pars opercularis, *IFGtr* IFG pars triangularis, *FusiG* fusiform gyrus, *MFG* middle frontal gyrus, *MidAntTemp* middle-anterior temporal lobe, *MidPostTemp* middle-posterior temporal lobe, *MTG* middle temporal gyrus, *PostTemp* posterior temporal lobe, *SFG* superior frontal gyrus, *SMG* supramarginal gyrus, *STG* superior temporal gyrus, *STS* superior temporal sulcus)

In this study we compared two different ROI analysis approaches. The first one employs functional partitions derived from a group level analysis which have been shown to include the language activity for the given paradigm of most individuals [4, 5] including the inferior frontal gyrus pars orbitalis, inferior frontal gyrus pars triangularis and opercularis, middle frontal gyrus, superior frontal gyrus, angular gyrus, anterior temporal lobe, middle-anterior temporal lobe, middle-posterior temporal lobe, posterior lobe, left fusiform gyrus, and right cerebellar hemisphere (Fig. 2a). Within these group-constrained functional partitions every participant’s individual fROI was defined in further steps.

The second approach uses standard anatomic regions automatically parceled and labeled [21]. Corresponding to the current knowledge about the location of language-sensitive cortical regions, [14] we selected the following aROIs for analysis: the inferior frontal gyrus pars opercularis, inferior frontal gyrus pars triangularis, middle frontal gyrus, supramarginal gyrus, angular gyrus, planum temporale, planum polare, superior temporal sulcus, and middle temporal gyrus (Fig. 2b).

Each ROI was analyzed in both hemispheres. Both, aROI masks and fROI masks were in the MNI standard space.

### Image Preprocessing and FMRI Data Analysis

The segmentation of the T1WI was carried out with FastSurfer [22]. Cortical labels from the automatic cortical parcellation were mapped to the automatic segmentation volume to assign the neuroanatomical labels of the Destrieux atlas [21]. MRI data was further analyzed with the FMRIB Software Library v6.0 using the FEAT processing pipeline [23, 24]. For preprocessing the fMRI data was motion corrected, spatial smoothed using a 5 mm full-width half-maximum Gaussian kernel, filtered with a high pass filter with a cut-off time of 90 s, co-registered to the anatomical reference image, and normalized to the MNI152 standard space T1-weighted average structural template image. For first-level analyses, experimental task and control blocks were convolved with the hemodynamic response function to evaluate the individual contrasts for sentences vs. nonword lists with the temporal derivatives as regressors of no interest. First-level statistic images were thresholded at *P* = 0.001 (uncorrected).

### Effect Size Extraction

**Fig. 3:**
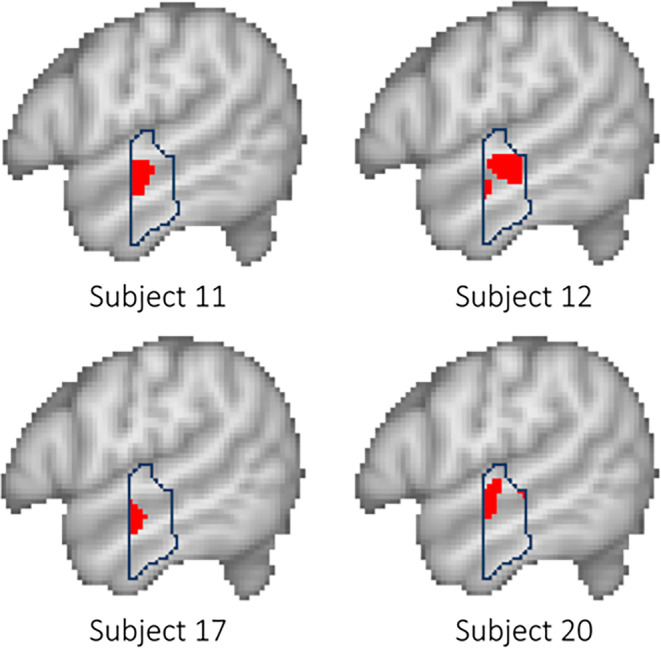
Examples for subject-specific functional ROIs for the left middle-anterior temporal cortex

To ensure data independence and avoid circularity, an across-runs cross-validation procedure was employed in the fROI analysis [4, 5, 25]. To this end, each subject’s activation map for the sentences > nonwords contrast was calculated using the data of three runs, leaving one run out. Of these three runs, the 10% of voxels with the highest *Z*‑score within a given partition were selected as that subject’s individual fROI, which was then applied on the independent data of the left-out run. This procedure was repeated across all possible combinations of the runs, and the responses were then averaged across the left-out runs to obtain a single response magnitude for a given region and subject. This approach has been outlined in prior work [4, 5] and we decided to use this approach based on the assumption that subject-specific functional localizers enhance the sensitivity and functional resolution of fMRI analyses [15–17]. This approach is particularly valuable in the context of language processing, where there is substantial inter-subject variability in the location and extent of functional activations. More specifically, if the selected top 10% of voxels truly reflect a reliable underlying signal, the mean percent signal change computed from the independent run should yield a meaningful effect size. Conversely, if the subject-specific fROI predominantly includes voxels driven by noise, the mean signal change in the independent run should approach zero, reflecting the absence of a replicable effect. Examples of subject-specific fROIs for the middle partition of the anterior temporal lobe can be found in Fig. 3.

For the aROI analysis we calculated each subject’s activation map across all four runs and extracted the response magnitude by averaging the signal intensity across all voxels in a given aROI mask.

Thus, at the core of this investigation lies the question of which ROI definition strategy most effectively supports robust and reliable quantification of language fMRI activations in individually tailored settings such as clinical language mapping. Our aim was to compare a recently proposed method for defining *group-constrained subject-specific* fROIs [4] with the aROI approach, which serves as an alternative for ROI-based quantitative analyses. This procedure introduces a size discrepancy between functional ROIs and anatomical ROIs (Supplementary Table 1). This difference in size reflects the underlying principles of each approach: anatomical ROIs cover broader cortical regions irrespective of activation strength, whereas functional ROIs are more spatially constrained, targeting only the most responsive voxels within a region.

Following statistical tests were performed on the percent blood oxygen level-dependent (BOLD) signal change values extracted from these fROIs and aROIs. The percent BOLD signal change value, also called effect size, was calculated by multiplying the parameter estimate map with the peak height of the hemodynamic response versus the baseline level of activity, divided by the mean signal of the run and multiplied by 100 [26].

### Group Statistics

To test whether the contrast sentences > nonwords led to an increased response in language-sensitive cortical regions, one-sample *t*-tests were calculated on effect sizes extracted for each ROI. Significance level was set to *P* = 0.00113, which is equivalent to a Bonferroni corrected alpha of 0.05.

To test whether one approach led to stronger effects than the other, the effect sizes of aROIs and fROIs were combined into the following regions: inferior frontal gyrus, middle frontal gyrus, angular gyrus and temporal lobe by calculating the average over the respective subregions (see Supplementary Tables 1 and 2). For each region, effect sizes were subsequently feed into four 2 × 2 repeated measures ANOVAs with the within-subjects factors Approach (fROI vs aROI) and Hemisphere (left vs. right). Furthermore, we determined laterality indices for each of the four regions by using the following formula [5]: laterality index = (left hemisphere ROI effect size – right hemisphere ROI effect size)/(left hemisphere ROI effect size + right hemisphere ROI effect size) and tested whether the fROI analysis yielded different laterality indices compared to the aROI analysis. To this end we calculated a 2 × 4 repeated measures ANOVA with the within-subjects factors Approach and ROI.

Finally, to assess the reliability of the two different analysis approaches, correlation analyses were conducted. We calculated between-sessions, within-subject Pearson correlation analysis for fROI and aROI effect sizes separately to compare the two approaches with regard to retest reliability. Fisher z‑transformation was applied to correlation coefficients and paired *t*-tests were conducted to compare the means of the transformed values. For the control analysis the correlation between the mean effect sizes of the first session of one subject and the second session of every other subject was calculated, for each participant. This between-session, between-subjects analysis was used to test whether the correlations within participants were higher than the correlations between participants between sessions, which would confirm the subject-specificity of the paradigm.

## Results

### Activation of Language-sensitive Regions

**Fig. 4:**
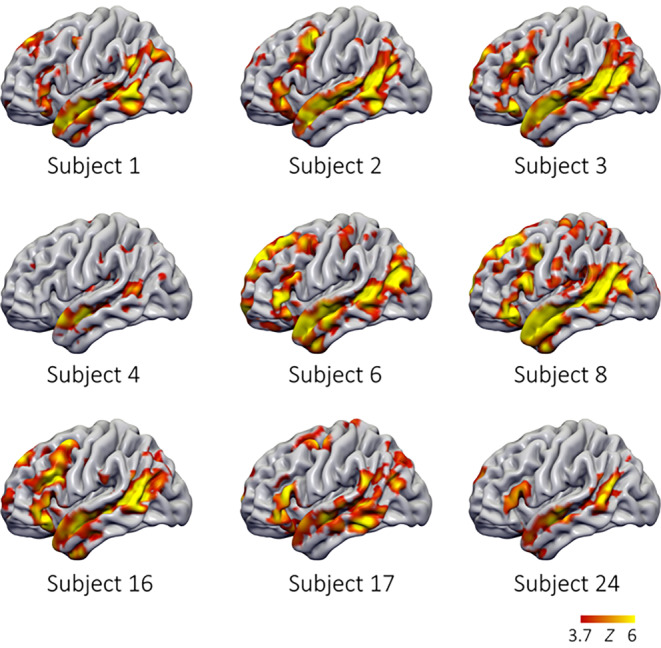
Examples of individual statistical maps for the contrast sentence > nonword. *Z* (Gaussianised T/F) statistic maps were thresholded using clusters determined by *Z* > 3.1 and a (corrected) cluster significance threshold of *P* = 0.05 (red-to-yellow 3.7 < *Z* < 6)

With our language paradigm, we generated a distinct activation in the known language regions in the left frontal, parietal and temporal lobes as well as in the right cerebellar hemisphere in each participant. Fig. 2a and b illustrate the topology of brain activity for the sentences > nonwords contrast. Individual example maps are shown in Fig. 4.

The grand average effect size was larger for fROIs (*M* = 0.28, *SD* = 0.14) than for aROIs (*M* = 0.05, *SD* = 0.09). One-sample t‑tests showed that all effect sizes in the fROI analysis were larger than zero (*P* < 0.00113, Fig. 2a). In the aROI analysis grand average effect sizes were also larger than zero, particularly in temporal regions. No significant positive percent BOLD signal changes were found for the inferior frontal gyrus pars opercularis, middle frontal gyrus and angular gyrus bilateral, the left supramarginal gyrus and planum polare, and the right inferior frontal gyrus pars triangularis and temporal pole (Fig. 2b).

### Comparison of the Analysis Approaches

**Fig. 5:**
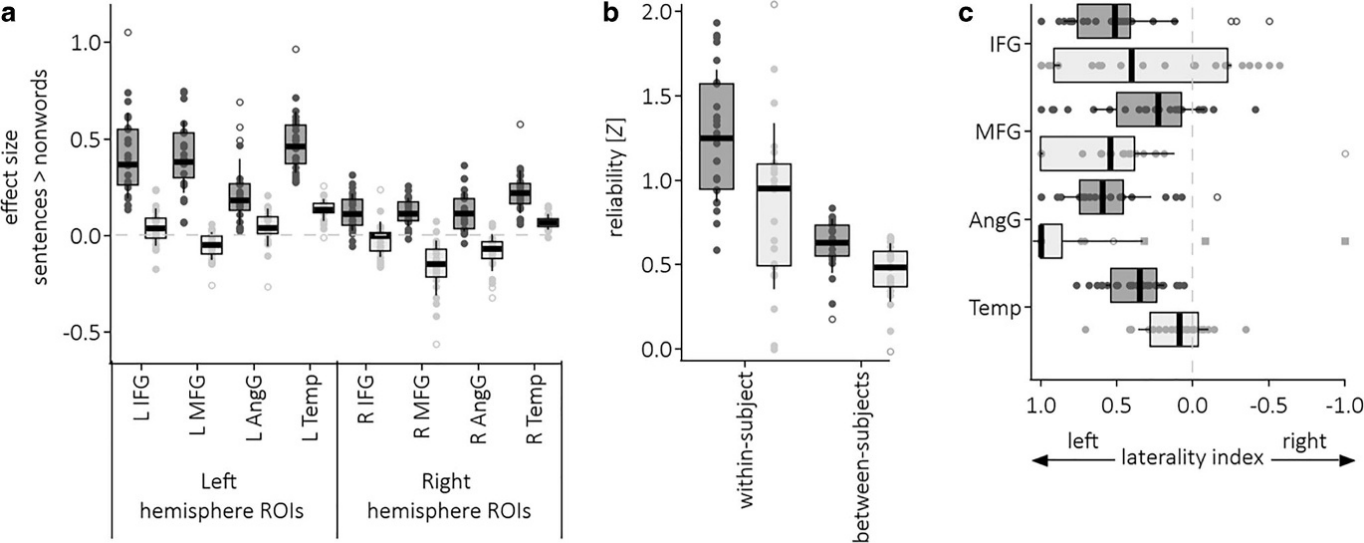
Comparison of the two analysis methods using fROIs (dark gray) and aROIs (light gray) in terms of (**a**) effectiveness, (**b**) retest reliability and (**c**) lateralization of language. (Abbreviations: *AngG* angular gyrus, *IFG* inferior frontal gyrus, *IFGorb* IFG pars orbitalis, *MFG* middle frontal gyrus, *Temp* temporal lobe)

Frontal, parietal and temporal language sensitive regions showed larger effect sizes in the fROI analysis than in the aROI analysis (Fig. 5a). All four ANOVAs indicated an effect of Approach, an effect of Hemisphere, and all but one ANOVA revealed an interaction between Approach and Hemisphere. Specifically, the ANOVA of the effect sizes of the inferior frontal gyrus revealed an interaction between Approach and Hemisphere, *F*(1, 23) = 37.8, *P* < 0.001, an effect of Approach, *F*(1, 23) = 144.2, *P* < 0.001, and an effect of Hemisphere, *F*(1, 23) = 38.6, *P* < 0.001. Temporal lobe analysis likewise revealed an interaction between Approach and Hemisphere *F*(1, 23) = 90.5, *P* < 0.001, an effect of Approach, *F*(1, 23) = 168.8, *P* < 0.001, and an effect of Hemisphere. *F*(1, 23) = 101.0, *P* < 0.001. Angular gyrus ANOVA revealed also an interaction between Approach and Hemisphere, *F*(1, 23) = 17.0, *P* < 0.001, an effect of Approach, *F*(1, 23) = 67.1, *P* < 0.001, and an effect of Hemisphere, *F*(1, 23) = 64.6, *P* < 0.001. Middle frontal gyrus analysis revealed an effect of Approach, *F*(1, 23) = 60.1, *P* < 0.001, and an effect of Hemisphere, *F*(1, 23) = 32.2, *P* < 0.001, but no interaction between Approach and Hemisphere *F*(1, 23) = 0.2, *P* = 0.69. All post-hoc *t*-test revealed significantly larger effect sizes for the fROI analysis compared to the aROI analysis (all *P* < 0.001, Bonferroni-corrected). Interactions indicated a stronger effect for left hemisphere ROIs compared to right hemisphere ROIs.

### Reliability of Analysis Approaches

The correlation coefficients within-subject between the two sessions were significantly higher for the fROI approach (*r* = 0.82, *SD* = 0.12, range 0.53 to 0.96) than for the aROI approach (*r* = 0.62; *SD* = 0.27; range −0.0001–0.96; *T* = 3.25, *P* = 0.004; Fig. 5b). The between-subjects between-sessions correlations were lower than the within-subject between-sessions correlation for both approaches, but the fROI analysis showed significantly higher correlations (*r* = 0.54, *SD* = 0.12, range 0.18 to 0.68) than the aROI analysis (*r* = 0.42, *SD* = 0.15, range −0.01 to 58; *T* = 3.26, *P* = 0.004) (Fig. 5b).

### Laterality Indices

Finally, we tested whether the two analysis approaches differed with respect to the determination of language lateralization (Fig. 5c). The ANOVA revealed no effect of Approach, *F*(1, 23) = 0.2, *P* = 0.65, but an effect of ROI, *F*(3, 69) = 11.5, *P* < 0.001, and an interaction between Approach and ROI, *F*(3, 69) = 10.5, *P* < 0.001. Post-hoc *t*-tests revealed a significant difference for the laterality indices in the temporal ROI with *T* = 4.1, *P* < 0.001, Bonferroni-corrected. Accordingly, fROI laterality indices were larger in the fROI (*M*_*effect size*_ = 0.38, *SD* = 0.19) than in the aROI analysis (*M*_*effect size*_ = 0.13, *SD* = 0.23). No differences occurred for the inferior frontal gyrus, middle frontal gyrus, and angular gyrus ROIs.

## Discussion

The German language task used in the current study robustly activated language-sensitive brain regions in each individual participant. The activation pattern showed reasonable overlap with a recently published probabilistic functional atlas of the core language network [3].

In clinical practice, it is often difficult to obtain robust, reliable and valid language network maps [2] or a meaningful estimate of effect sizes and language lateralization in a reasonable amount of time. This problem is also caused by the need to set statistical thresholds and correct for multiple testing, a problem that can be minimized by the use of predefined ROIs [12]. Anatomical ROIs are readily available but tend to be large and may dilute the signal, whereas functional ROIs are more spatially specific but harder to obtain, as they require additional scanning runs. Here we show, that the use of subject-specific functional ROIs provides more precise and informative activation patterns compared to standard anatomical ROIs. The percent BOLD signal change determined with the fROI approach was up to nine times higher compared to the aROI approach, see for example average effect sizes of the left inferior frontal gyrus (Fig. 5a). One reason for this improvement of signal estimation is that the fROI approach accounts for individual location of the peak activation in respective eloquent regions. In contrast, the aROIs are larger than the actual speech-sensitive region, so the estimate of percent signal change is likely contaminated by voxels that contain noise rather than signal.

The advantage of the fROI approach was also evident in the test-retest reliability analysis. Individual response profiles obtained using the fROI approach showed a good to excellent reliability across the two sessions, while for the aROI approach reliability was poor to good. The previous study reported satisfying reliability for the current language paradigm in a different language, when comparing signal intensity across all voxels in the selected functional partitions [5]. Here we add data demonstrating that the actual individual activation profiles are reliably reproducible with this language task and analysis approach, a highly relevant aspect for preoperative fMRIs.

Between-subject between-session correlations were moderate to poor for both analysis approaches (Fig. 5b) indicating the individual characteristics of each participants activation profile. Still, the measurable consistency between participants aligns with the assumption that the core language network is a spatially distributed set of high functionally integrated brain regions, whose topology is similar [3].

Language lateralization estimates differed for the temporal ROI. However, for several ROIs the effect sizes in the aROI analysis were not significantly different from zero, which will likely interfere with a reliable estimate of language lateralization. Building quotients on the basis of very small values will lead to far too large effect sizes [5] (e.g., angular gyrus, middle frontal gyrus, Fig. 5c). Therefore, this finding demonstrates that it is necessary to first evaluate the robustness of an activation before calculating laterality quotients which are prone to outliers and distort the finding.

One of the key aims of clinical language fMRI is not to exhaustively localize all regions involved in language processing, but to determine hemispheric language dominance. This is particularly important for identifying atypical right or bilateral language representation, which is relatively common in patients with early left-hemisphere injury [27, 28], or for confirming expected left dominance, especially in healthy right-handers, where left-hemisphere dominance is typical, though right-hemisphere dominance occurs more often than previously assumed [29, 30]. In such cases, additional methods like the Wada test or electrocortical stimulation may be required for presurgical planning. Nonetheless, fMRI remains a valuable tool for detecting atypical dominance, assessing the risk of postoperative language deficits, and informing electrode placement for electrocortical stimulation. With this clinical objective in mind, our study aims to support the development of more practical and reliable mapping protocols for routine lateralization assessment.

Finally, we consider limitations. In contrast to previous studies which used at least 8‑word sentences or nonword lists [4, 5] here we used 4‑word conditions to make the task more feasible for clinical populations who may already have cognitive impairment. Future studies could evaluate the suitability of this approach in patient groups with neurological disorders. Moreover, whereas prior work assessed robustness and reliability of their paradigm with 7 to 8 runs, the current study was limited to 4 runs. Therefore, the effect sizes are comparably smaller and may be improved for better interpretation of activation in every ROI. Nevertheless, the laterality is clearly visible, which is one of the primary questions in the context of preoperative planning. In the clinical routine a tradeoff between time investment and quality of measuring result is ubiquitous and should be considered for finding a compromise when implementing a new method.

Previous work has shown that language tasks recruit both domain-specific and domain-general networks, with notable overlap in the frontal and parietal cortices [31]. To control for this potential confound, we adopted a carefully matched control task. Specifically, participants read lists of pronounceable nonwords that were matched to the sentence stimuli in terms of key linguistic features, including syllable count, phonetic complexity, and phonotactic structure. This control condition was used not only to engage the visual system but also to elicit cognitive processes such as verbal working memory and attentional control. Additionally, both the experimental and control tasks required participants to make a decision and respond via button press, thereby equating cognitive and motor demands across conditions. The resulting activation patterns can therefore be attributed to language-specific processing, as the control task effectively accounted for non-linguistic demands such as visual perception, working memory load, and motor response preparation. This approach has been extensively validated in prior fMRI research [32–35].

Current recommendations propose the usage of different paradigms in order to cover the different aspects of language processing [2]. The language task used in this study enables the mapping of semantic language activity with reduced activation of the multiple demand network by usage of the sentence > nonwords contrast [32–35] and therefore potentially complements the subset of recommended language paradigms for presurgical assessment. Further studies are needed to examine this approach in the clinical setting and could aim for the enlargement of the eligible patient population by testing further adaptions of the language paradigm, e.g., regarding number of runs needed, minimum length of each run or timing of the block design to adjust to individual preconditions.

## Conclusion

The investigated language task led to a robust and reliable activation of eloquent regions in the individual subject. The choice of analysis significantly influenced the outcome in terms of percent BOLD signal change, test-retest reliability, and language laterality. Subject-specific fROIs performed better than standard anatomical ROIs and are to be preferred in clinical routine for fMRI paradigms with short measurement time and low signal change.

## Supplementary Information

Supplementary information includes Supplementary Tables 1 and 2.

## Abbreviations

aROI: anatomical ROI
BOLD: blood oxygen level-dependent
fMRI: functional MRI
fROI: functional ROI
