# Supplementary material for: Subject-specific Functional ROIs Enhance Reliability in Language FMRI
Source: Clin Neuroradiol. 2025 Jul 9;35(4):775–83. doi: 10.1007/s00062-025-01534-3 (PMC12552277; doi:10.1007/s00062-025-01534-3)
Supplement: Supplementary file 1 — Supplementary information includes Supplementary Tables 1 and 2. [file 62_2025_1534_MOESM1_ESM.docx]

**Subject-specific functional ROIs enhance reliability in language fMRI**

Julia My Van Kube, Luisa Katrin Thomas, Peter Dechent, Christian Heiner Riedel, Nicole E. Neef

**Supplementary information**

**Roi sizes**

For the *Group-Constrained Subject-Specific* (GCSS) fROI approach, ROI sizes are comparable across the left and right hemispheres and are therefore reported only once in Supplementary Table 1. The column labeled "GC volumes" presents the volumes of the group-constrained functional partitions, which are publicly available from the repository provided by the Fedorenko lab [1,2]. These partitions are visualized on the 3D-rendered left hemisphere of the standard brain template (Figure 2A, upper left panel). The column labeled **"SS volumes"** shows the 10% subset volumes derived from these partitions, representing the actual fROI volumes used for effect size quantification. Example fROI masks are illustrated in Figure 3.

**Supplementary Table 1** ROI size in mm³

| **ANOVA**  ROI | **Group-Constrained (GC) Subject-Specific (SS) ROI** | | | **Anatomtical ROI** | |  |
| --- | --- | --- | --- | --- | --- | --- |
|  | fROI | GC | SS (top 10%) | aROI | left | right |
|  | IFGorb | 23408 | 2341 |  |  |  |
| **IFG** | **IFG** | **27752** | **2775** | **IFGop** | **2537** | **2471** |
|  |  |  |  | **IFGtr** | **1223** | **1289** |
| **MFG** | **MFG** | **24288** | **2429** | **MFG** | **5913** | **5565** |
|  | SFG | 16568 | 1657 |  |  |  |
| **AngG** | **AngG** | **17840** | **1784** | **AngG** | **3199** | **2883** |
| **Temp** | **AntTemp** | **15648** | **1565** | **SMG** | **4086** | **3913** |
|  | **MidAntTemp** | **16792** | **1679** | **STG Plan tempo** | **1484** | **1055** |
|  | **MidPostTemp** | **38456** | **3846** | **STG Lateral** | **4484** | **3906** |
|  | **PostTemp** | **19184** | **1918** | **STG Plan polar** | **1578** | **1463** |
|  |  |  |  | **STS** | **4652** | **6040** |
|  |  |  |  | **Pole temporal** | **4131** | **4475** |
|  |  |  |  | **MTG** | **4906** | **5501** |
|  | Cereb | 10584 | 1058 |  |  |  |

AngG = angular gyrus; AntTemp = anterior temporal lobe; Cereb = Cerebellum; IFG = inferior frontal gyrus; IFGorb = IFG pars orbitalis; IFGop = IFG pars opercularis; IFGtr = IFG pars triangularis; FusiG = fusiform gyrus; MFG = middle frontal gyrus; MidAntTemp = middle anterior temporal lobe; MidPostTemp = middle posterior temporal lobe; MTG = middle temporal gyrus; PostTemp = posterior temporal lobe, SFG = superior frontal gyrus; SMG = supramarginal gyrus; STG = superior temporal gyrus; STS = superior temporal sulcus

Supplementary Table 2 lists the ROI volumes used for effect size calculations in the comparison of the fROI and aROI approaches within the ANOVAs. Effect sizes were pooled for this analysis to enable statistical testing in cortical regions particularly relevant for presurgical neuroradiological evaluation of language function.

**Supplementary Table 2** ROI size in mm³ for the ANOVA

|  | fROI | aROI |  | Top 10% aROI | fROI | aROI | Top 10% aROI |
| --- | --- | --- | --- | --- | --- | --- | --- |
|  | *left* | | | | *right* | | |
| IFG | 2775 | 3760 |  | 135 | 2775 | 3760 | 135 |
| MFG | 2429 | 5913 |  | 243 | 2429 | 5565 | 229 |
| AngG | 1784 | 3199 |  | 179 | 1784 | 2883 | 162 |
| Temp | 9008 | 21235 |  | 236 | 9008 | 22440 | 249 |

AngG = angular gyrus; IFG = inferior frontal gyrus; MFG = middle frontal gyrus; Temp = temporal lobe regions

**References**

1. Fedorenko E, Hsieh P-J, Nieto-Castañón A, Whitfield-Gabrieli S, Kanwisher N. New Method for fMRI Investigations of Language: Defining ROIs Functionally in Individual Subjects. J Neurophysiol. 2010;104:1177–94.

2. Mahowald K, Fedorenko E. Reliable individual-level neural markers of high-level language processing: A necessary precursor for relating neural variability to behavioral and genetic variability. Neuroimage. 2016;139:74–93.
